# Supplementary material for: Disruption of recombination machinery alters the mutational landscape in plant organellar genomes
Source: G3 (Bethesda). 2025 Feb 13;15(4):jkaf029. doi: 10.1093/g3journal/jkaf029 (PMC12005158; doi:10.1093/g3journal/jkaf029)
Supplement: jkaf029_Supplementary_Data [file jkaf029_supplementary_data.zip › Supplemental_Material_G3-2024-405483.docx]

**SUPPLEMENTAL FIGURES**


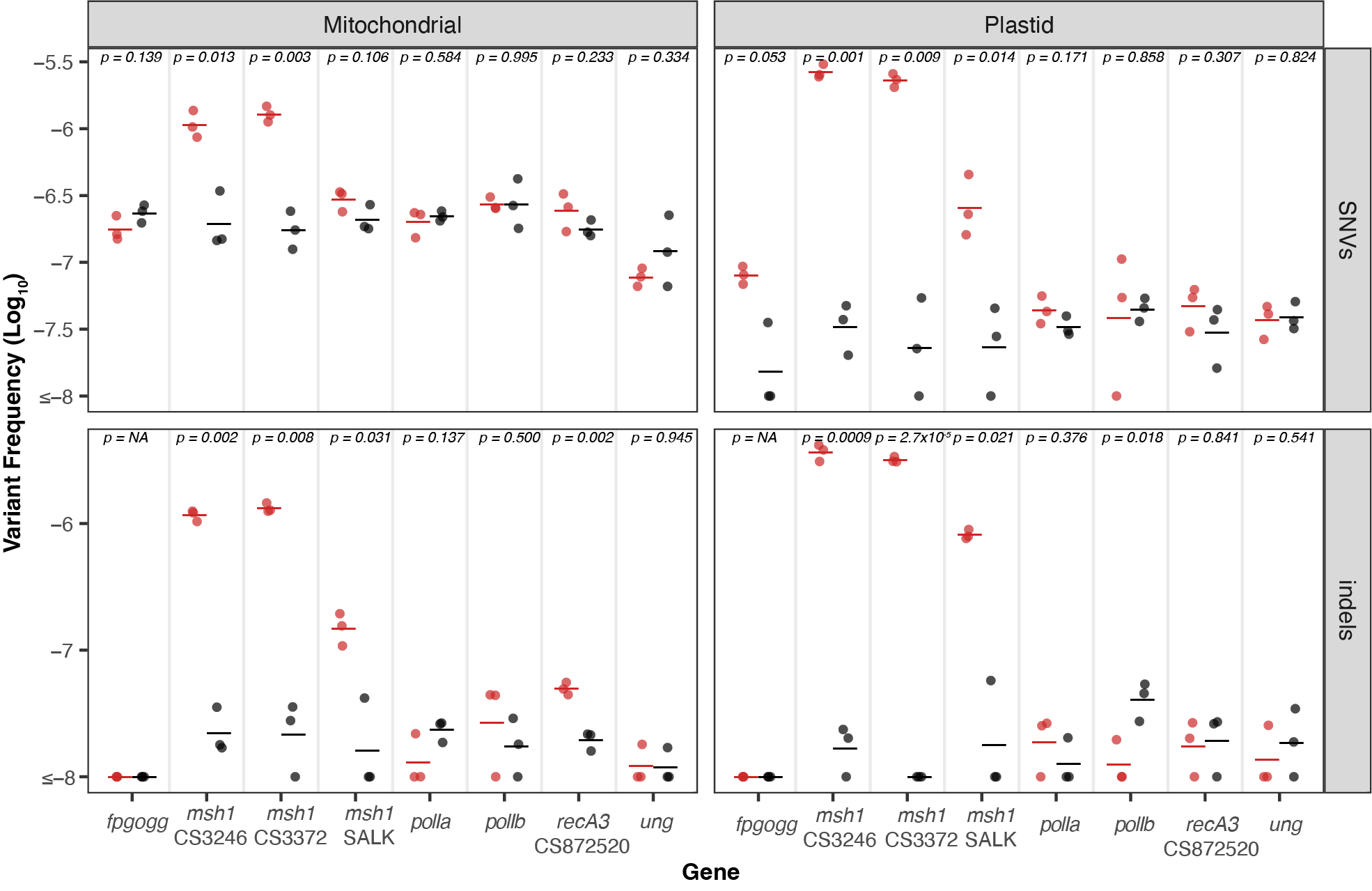


Figure S1. *De novo* point mutations measured with Duplex Sequencing from data generated in Wu *et al.* 2020. For each gene of interest (x-axis) mutant lines are plotted in red and matched WT controls are plotted in black. The individual biological replicates are plotted as circles, and group averages are plotted as dashes. Panels separate the data by genome (left column: Mitochondria and right column: Plastid) and by point mutation type (top row: SNVs and bottom row: indels). The y-axis shows the log-transformed SNV frequencies (total SNVs/total DCS coverage). P-values show the result of a two-tailed *t*-test comparing WT vs mutant mutation frequencies for each gene of interest. We found significant increases in SNV and indel frequencies in the *msh1* ­CS3246 and *msh1* CS3372 mutants (both genomes) but the *msh1* SALK046763 mutant, which is not a complete knockout of the *msh1* gene (Wu *et al.,* 2020) had weaker effects. In addition, we note that this *recA3* null allele is different from the *recA3* null allele that was reported in the new dataset, but both yielded similar results: significant indel and weakly significant SNV increases in mtDNA of the *recA3* mutant. Also note the marginally significant difference in *fpg*/*ogg* plastid SNVs is explained by just 5 SNVs in mutants and a single SNV in the WT controls, which we do not consider to be a biologically meaningful difference.

*
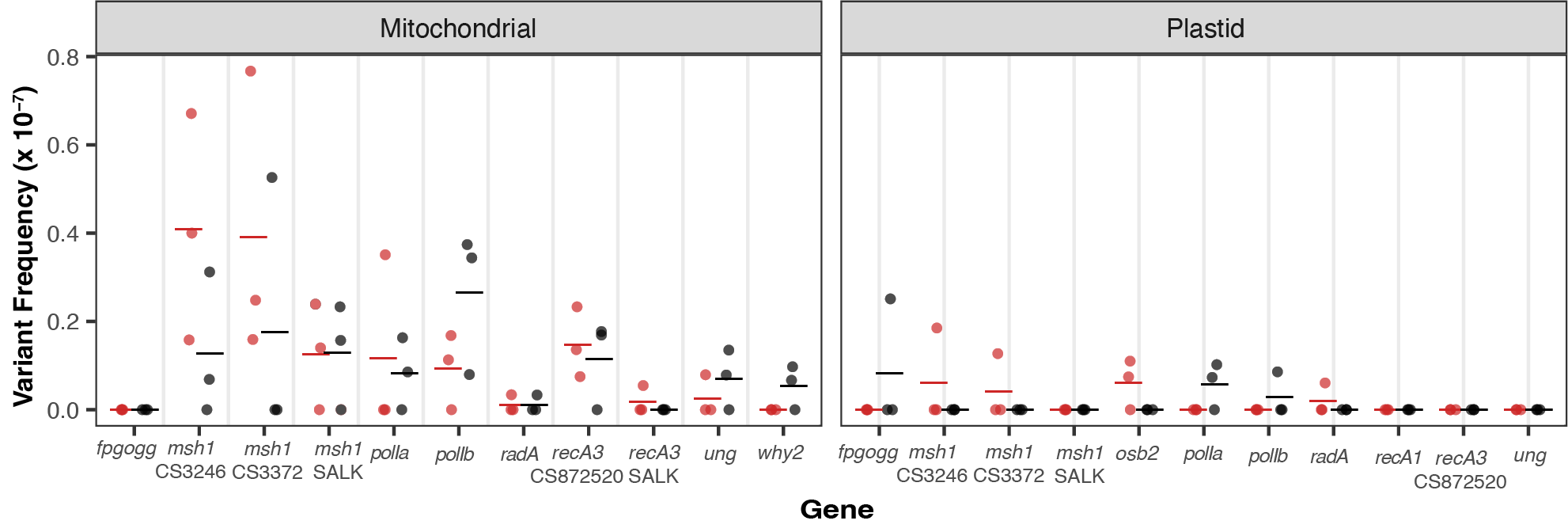
*

Figure S2. Dinucleotide mutations measured with Duplex Sequencing. For each gene of interest (x axis) mutant lines are plotted in red, and matched WT controls are plotted in black. The individual biological replicates are plotted as circles, and group averages are plotted as dashes. Panels divide the data by mitochondrial and plastid. We performed Wilcoxon rank sum tests to look for differences between mutant and matched WT controls and all p-values were > 0.05. Note that *recA3* CS872520 dataset was generated in Wu *et al.* (2020), and the *recA3* SALK 146388 dataset was generated in this study.


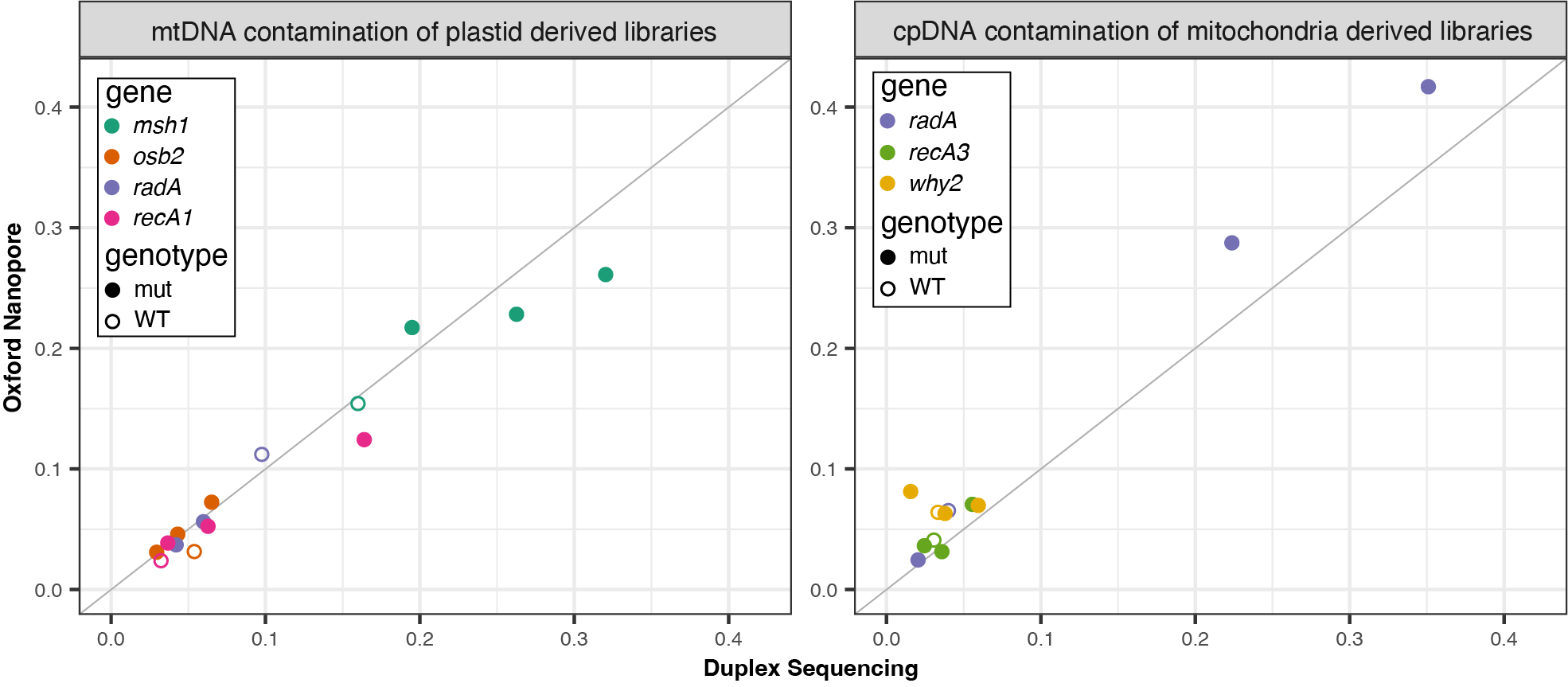


Figure S3. Correlation of cross-organelle contamination in Oxford Nanopore and Duplex Sequencing libraries. Contamination is calculated as the number of contaminating reads in the read alignments divided by the total number of organellar alignments. The different mutant lines are colored according to the figure legend with mutant replicates plotted using closed circles and matched WT controls plotted with open circles. The 1:1 diagonal line is shown in gray. Though the level of contamination varies between different DNA samples (for example mtDNA contamination is higher in the plastid derived *msh1* libraries) the contamination levels are generally similar irrespective of sequencing technique. Note, For the *msh1* mtDNA analysis, we relied exclusively the plastid-derived *msh1* samples, and for the *radA* mtDNA analysis, we used a combination of the low coverage *radA* mitochondrial samples and the plastid *radA* samples (see main text).

Figure S4. Median read length cross-organelle contaminating and native reads in the plastid and mitochondrial derived nanopore libraries. The different mutant lines are colored according to the figure legend with mutant replicates plotted using closed circles and matched WT controls plotted with open circles. The 1:1 diagonal line is show in gray. Note, For the *msh1* mtDNA analysis, we relied exclusively the plastid-derived *msh1* samples, and for the *radA* mtDNA analysis, we used a combination of the low coverage *radA* mitochondrial samples and the plastid *radA* samples (see main text).


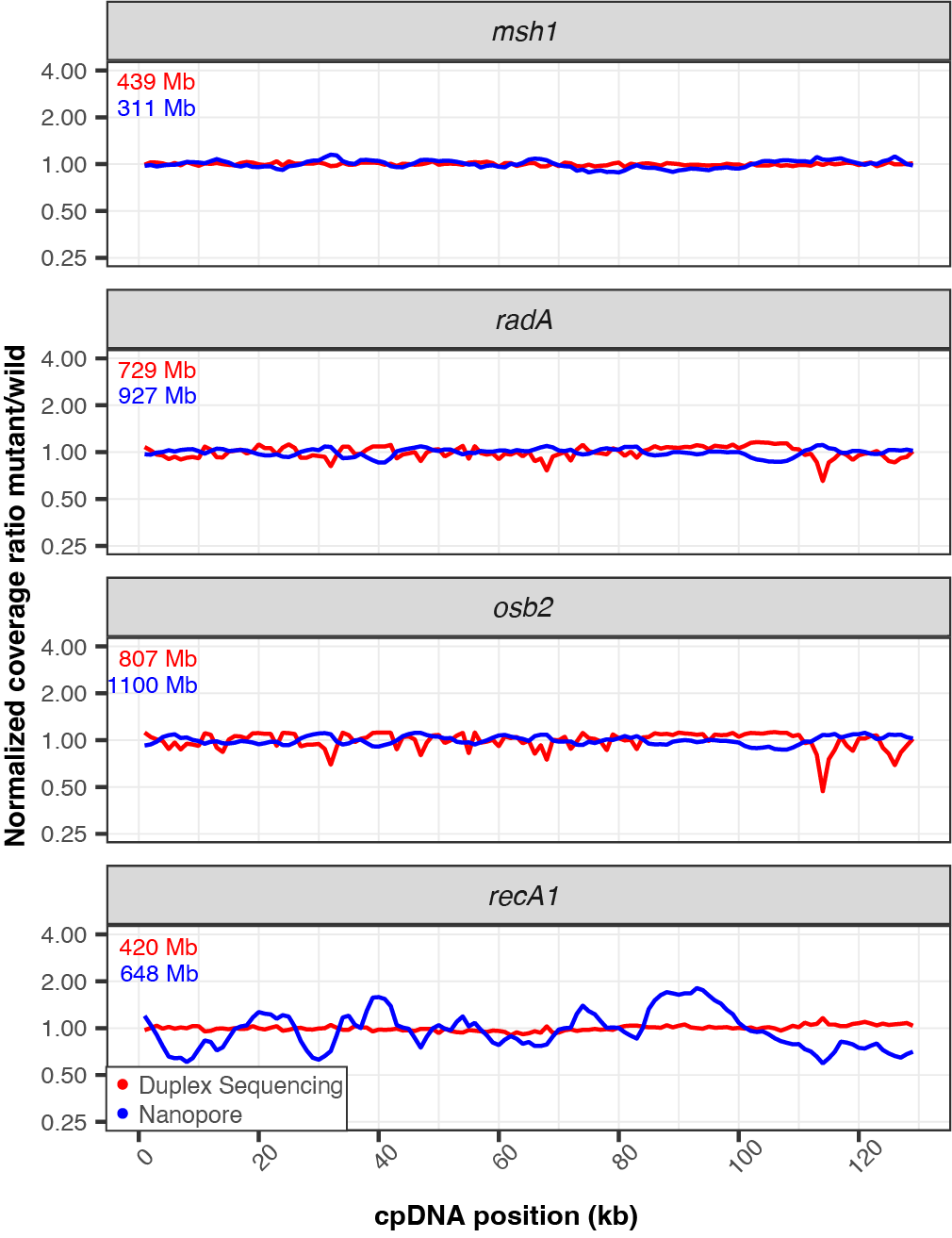


Figure S5. Normalized coverage of plastid genomes in mutant lines of interest . Coverage of each Duplex Sequencing (red) or nanopore (blue) library was calculated in 1000-bp windows. Mutant coverage was pooled and divided by WT coverage and the resulting ratios were normalized to 1 for plotting. The total amount of sequencing data used to generate each plot is shown in the top left corner of each panel (red=Duplex Sequencing and blue=nanopore) and is included to highlight the instances where disagreement between the Duplex Sequencing and nanopore lines may be explained by increased variance in the nanopore sample due to lower mtDNA coverage. To see the coverage of the individual replicates see Fig S8 and S9.


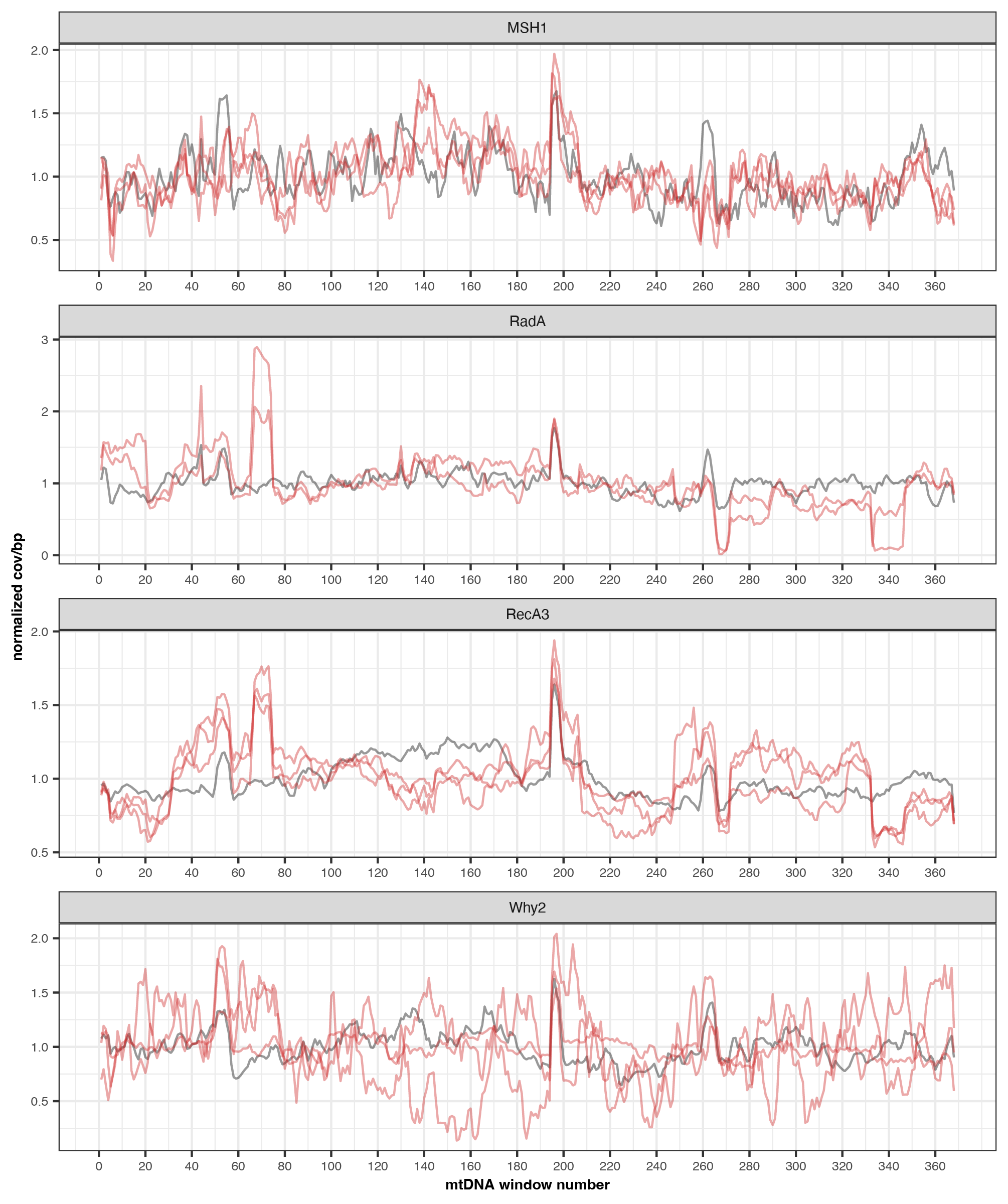


Figure S6. Normalized coverage of the individual nanopore mtDNA replicates (used to generate Fig. 8). The red and black lines show the normalized coverage of the mutant replicates and the matched WT control, respectively. Note that variation in the *why2* mutants is likely due to extremely low coverage in these samples (average coverage per bp of 157.3, 6.5 and 7.0 in mutant replicates 1, 2 and 3, respectively).


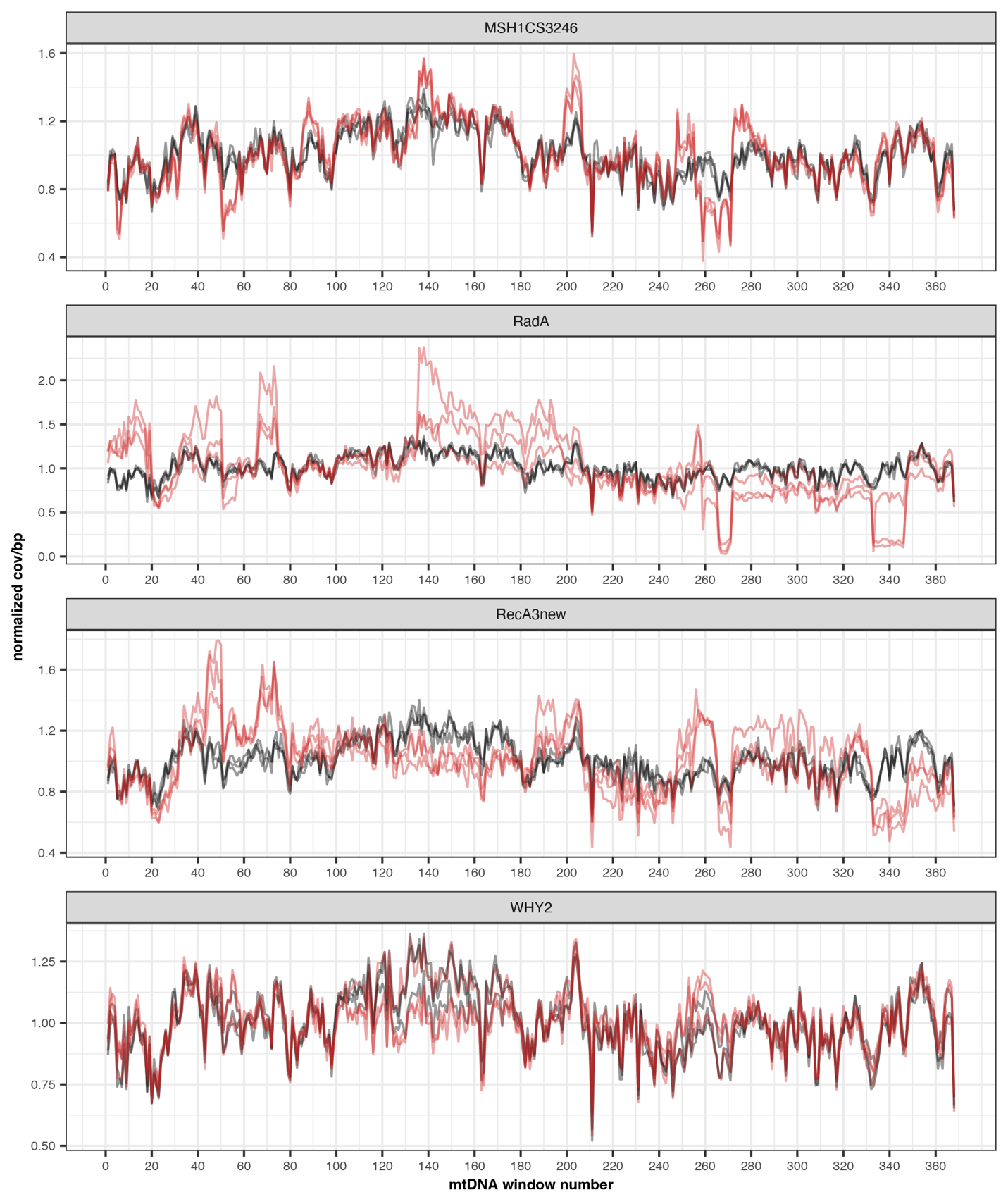


Figure S7. Depth of coverage of the individual Duplex Sequencing mtDNA replicates (used to generate Fig. 8). The red and black lines show the normalized coverage of the mutant replicates and the matched WT control, respectively.


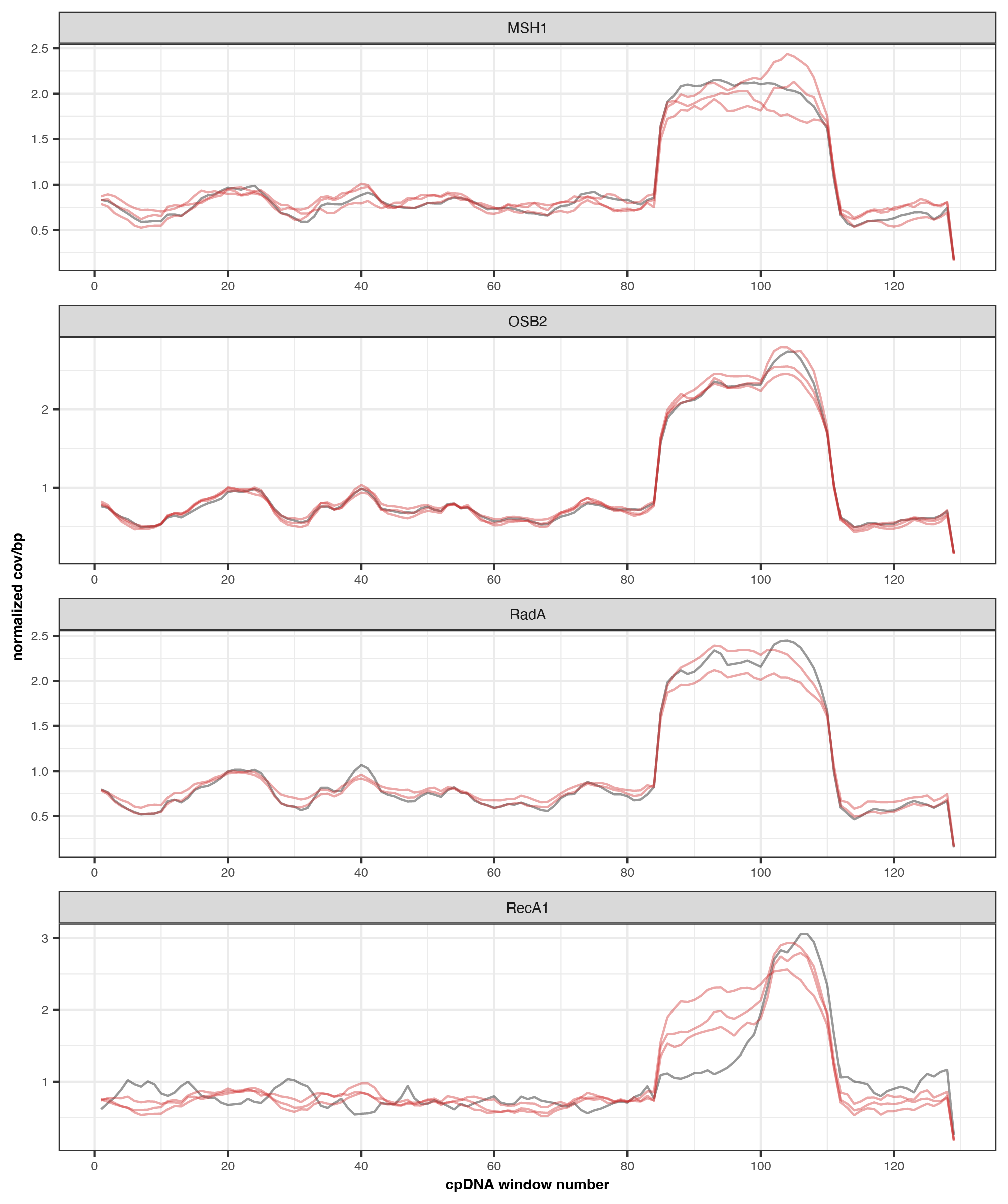


Figure S8. Normalized of coverage of the individual nanopore cpDNA replicates (used to generate Fig. 8). The red and black lines show the normalized coverage of the mutant replicates and the matched WT control, respectively. Note that the spike in coverage at ~84-112 kb results from the large inverted repeat, since these reads were mapped noncompetitively with minimap2 (see methods). The second copy of the inverted repeat was omitted for plotting.


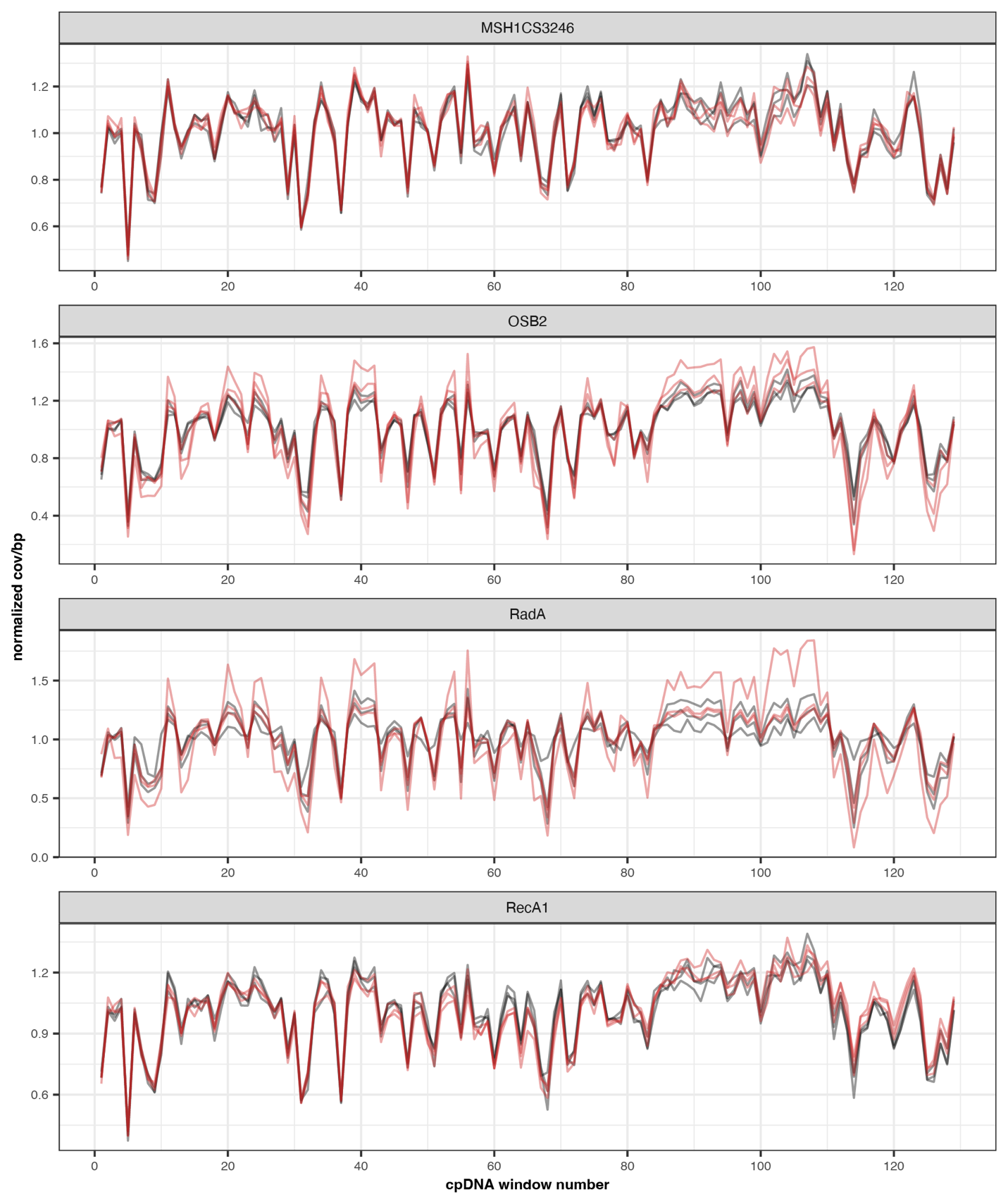


Figure S9. Normalized coverage of the individual Duplex Sequencing cpDNA replicates (used to generate Fig. 8). The red and black lines show the normalized coverage of the mutant replicates and the matched WT control, respectively. Note these reads were mapped to a full length cpDNA but the second large inverted repeat was omitted for plotting.

**SUPPLEMENTAL TABLES**

**Table S1**. Mutant lines used in this study and primers to verify plant genotype

| **Gene** | **Salk line (all from ABRC)** | **Locus** | **Forward Primer Wild** | **Forward Primer Mutant (LBb1.3)** | **Reverse Primer Wild and Mutant** |
| --- | --- | --- | --- | --- | --- |
| *radA* | SALK_097880 | AT5G50340 | TTTCACTTATCGAGCCAGAGC | ATTTTGCCGATTTCGGAAC | ATGCCATAATGCTTTTTGCTG |
| *recA1* | SALK_072979 | AT1G79050 | TAGGGTGAGATTGGAATGCAG | ATTTTGCCGATTTCGGAAC | AAGAGCTGCTGCTCATCAAAG |
| *recA3* | SALK_146388 | AT3G10140 | CGTTTGGTCAGTTGAAGCTTC | ATTTTGCCGATTTCGGAAC | CTCCACAAGTCACTTCTTCGG |
| *osb2* | SALK_061852 | AT4G20010 | AGCGTGAAAGGTGAGACGTT | ATTTTGCCGATTTCGGAAC | GGGAAATAACAGTACCAGCCC |
| *why2* | SALK_118900 | AT1G71260 | CAGGAAGTCACTGTCAGTTAAGC | ATTTTGCCGATTTCGGAAC | ACCCATGATTTAGAAGTCTTAGAGAGG |

**Table S2.** Oxford Nanopore sequencing yields for each of the three runs

| **Sample** | **Sequencing run** | **Read count** | **Long reads count (>500bp)** | **Total yield (Mb)** |
| --- | --- | --- | --- | --- |
| plastid_recA1_wild_1 | 1 | 224238 | 131719 | 412.21 |
| plastid_recA1_mut_1 | 1 | 82051 | 39041 | 121.45 |
| plastid_recA1_mut_2 | 1 | 74341 | 32851 | 130.77 |
| plastid_recA1_mut_3 | 1 | 72833 | 41137 | 167.30 |
| plastid_radA_wild_1 | 1 | 127499 | 85335 | 307.34 |
| plastid_radA_mut_1 | 1 | 111390 | 72395 | 297.02 |
| plastid_radA_mut_3 | 1 | 186393 | 119090 | 540.66 |
| plastid_osb2_wild_3 | 1 | 101793 | 66081 | 239.62 |
| plastid_osb2_mut_1 | 1 | 143806 | 92534 | 407.94 |
| plastid_osb2_mut_2 | 1 | 103151 | 74649 | 349.70 |
| plastid_osb2_mut_3 | 1 | 109492 | 63761 | 260.72 |
| plastid_msh1_wild_3 | 1 | 45501 | 29533 | 126.16 |
| plastid_msh1_mut_1 | 1 | 36518 | 24441 | 111.72 |
| plastid_msh1_mut_2 | 1 | 46533 | 26330 | 97.91 |
| plastid_msh1_mut_3 | 1 | 47757 | 32369 | 153.13 |
| mitochondrial_recA3_wild_1 | 2 | 8481 | 6019 | 56.73 |
| mitochondrial_recA3_mut_1 | 2 | 1442 | 813 | 8.70 |
| mitochondrial_recA3_mut_2 | 2 | 20256 | 14861 | 101.19 |
| mitochondrial_recA3_mut_3 | 2 | 13261 | 8069 | 52.79 |
| mitochondrial_radA_wild_3 | 2 | 2119 | 766 | 4.64 |
| mitochondrial_radA_mut_1 | 2 | 13790 | 6079 | 22.39 |
| mitochondrial_radA_mut_2 | 2 | 3675 | 154 | 0.90 |
| mitochondrial_radA_mut_3 | 2 | 1384 | 681 | 6.32 |
| mitochondrial_why2_wild_1 | 2 | 4720 | 2629 | 20.01 |
| mitochondrial_why2_mut_1 | 2 | 9965 | 6992 | 50.01 |
| mitochondrial_why2_mut_2 | 2 | 1112 | 411 | 3.34 |
| mitochondrial_why2_mut_3 | 2 | 1279 | 287 | 2.78 |
| mitochondrial_MSH1_wild_1 | 2 | 931 | 95 | 0.52 |
| mitochondrial_MSH1_mut_1 | 2 | 959 | 151 | 0.64 |
| mitochondrial_MSH1_mut_2 | 2 | 925 | 50 | 0.42 |
| mitochondrial_MSH1_mut_3 | 2 | 471 | 34 | 0.22 |
| mitochondrial_recA3_wild_1 | 3 | 16270 | 12616 | 120.48 |
| mitochondrial_recA3_mut_1 | 3 | 1684 | 1028 | 11.65 |
| mitochondrial_recA3_mut_2 | 3 | 13791 | 10705 | 97.58 |
| mitochondrial_recA3_mut_3 | 3 | 13488 | 9944 | 82.53 |
| mitochondrial_radA_wild_3 | 3 | 869 | 650 | 3.98 |
| mitochondrial_radA_mut_1 | 3 | 18460 | 13217 | 53.96 |
| mitochondrial_radA_mut_2 | 3 | 393 | 44 | 0.19 |
| mitochondrial_radA_mut_3 | 3 | 1017 | 500 | 5.02 |
| mitochondrial_why2_wild_1 | 3 | 3596 | 2496 | 18.99 |
| mitochondrial_why2_mut_1 | 3 | 6147 | 3618 | 26.85 |
| mitochondrial_why2_mut_2 | 3 | 1507 | 85 | 0.62 |
| mitochondrial_why2_mut_3 | 3 | 508 | 92 | 0.77 |

Note that, for the *radA* mtDNA analysis, we averaged structural variant frequencies and coverages across the mitochondrially and plastid-derived samples, while for *msh1,* we relied entirely on the plastid-derived samples and did not investigate the mitochondrially derived samples, which had extremely low yield.

**Table S3.** Duplex read-pairs and organellar genome coverage

| **Sample** | **Count of read-pairs (2x150)** | **Organellar coverage per bp** |
| --- | --- | --- |
| mitochondrial_rada_mut_1 | 75863350 | 297.9 |
| mitochondrial_rada_mut_2 | 64771627 | 281.8 |
| mitochondrial_rada_mut_3 | 139195192 | 803.4 |
| mitochondrial_rada_wild_1 | 70847671 | 127.8 |
| mitochondrial_rada_wild_2 | 61998713 | 246.9 |
| mitochondrial_rada_wild_3 | 127161861 | 816.4 |
| mitochondrial_reca3_mut_1 | 43472074 | 94.2 |
| mitochondrial_reca3_mut_2 | 44312097 | 229.3 |
| mitochondrial_reca3_mut_3 | 59128403 | 497.3 |
| mitochondrial_reca3_wild_1 | 62354817 | 238.2 |
| mitochondrial_reca3_wild_2 | 54311915 | 183.2 |
| mitochondrial_reca3_wild_3 | 40734051 | 144.8 |
| mitochondrial_why2_mut_1 | 63375069 | 338.0 |
| mitochondrial_why2_mut_2 | 76906783 | 284.9 |
| mitochondrial_why2_mut_3 | 76221972 | 292.6 |
| mitochondrial_why2_wild_1 | 68231709 | 279.8 |
| mitochondrial_why2_wild_2 | 81396138 | 379.9 |
| mitochondrial_why2_wild_3 | 86880259 | 408.4 |
| plastid_osb2_mut_1 | 47505179 | 1176.6 |
| plastid_osb2_mut_2 | 54307516 | 870.8 |
| plastid_osb2_mut_3 | 59415250 | 898.6 |
| plastid_osb2_wild_1 | 59542949 | 1132.8 |
| plastid_osb2_wild_2 | 69408084 | 889.7 |
| plastid_osb2_wild_3 | 67727784 | 668.6 |
| plastid_rada_mut_1 | 76116128 | 1174.4 |
| plastid_rada_mut_2 | 68615282 | 871.7 |
| plastid_rada_mut_3 | 45985626 | 1068.7 |
| plastid_rada_wild_1 | 53480887 | 234.2 |
| plastid_rada_wild_2 | 46684396 | 954.5 |
| plastid_rada_wild_3 | 46190084 | 776.4 |
| plastid_reca1_mut_1 | 66804365 | 543.7 |
| plastid_reca1_mut_2 | 38396319 | 594.8 |
| plastid_reca1_mut_3 | 30645358 | 299.3 |
| plastid_reca1_wild_1 | 37377457 | 598.2 |
| plastid_reca1_wild_2 | 32420159 | 543.5 |
| plastid_reca1_wild_3 | 33351491 | 331.1 |

**Table S4**. SNV frequencies of various libraries used to test if changes to our library preparation or sequencing protocol explain reduction in SNV frequencies in the newly generated WT libraries.

| **DNA sample** | **Library preparation** | **Blue Pippin** | **Sequence generated** | **SNV frequency** |
| --- | --- | --- | --- | --- |
| Mitochondrial extraction, WT replicate 3 from *msh1-*CS3246 line (Wu *et al.* 2020) | New library (see methods) | Yes | This study (updated NovaSeq 6000 chemistry) | 1.57´10^-7^ |
| Mitochondrial extraction, WT replicate 3 from *msh1-*CS3246 line (Wu *et al.* 2020) | Old library (see Wu *et al.,* 2020) | No | Wu *et al,.* 2020 (original NovaSeq 6000 chemistry) | 1.39´10^-7^ |
| Mitochondrial extraction, WT replicate 3 from *msh1-*CS3246 line (Wu *et al.* 2020) | Old library (see Wu *et al.,* 2020) | No | This study (updated NovaSeq 6000 chemistry) | 1.47´10^-7^ |
| Mitochondrial extraction, WT replicate 2 from *msh1-*CS3246 line (Wu *et al.* 2020) | Old library (see Wu *et al.,* 2020) | Yes | This study (updated NovaSeq 6000 chemistry) | 1.97´10^-7^ |
| Mitochondrial extraction, WT replicate 2 from *msh1-*CS3246 line (Wu *et al.* 2020) | Old library (see Wu *et al.,* 2020) | No | Wu *et al,.* 2020 (original NovaSeq 6000 chemistry) | 1.36´10^-7^ |

**Table S5.** Results from Kruskal-Wallis test comparing SNV frequencies among genomic regions in WT and *msh1* mutant data from Wu *et al.,* (2020)

| **Sample** | **Kruskal-Wallis chi-squared value** | **p-value** |
| --- | --- | --- |
| *msh1* mitochondria | 6.03 | 0.19 |
| *msh1* plastid | 5.47 | 0.24 |
| WT mitochondria | 6.66 | 0.15 |
| WT plastid | 11.35 | 0.02 |

**Table S6.** Sequencing depth per bp (calculated with bedtools depth) of samples in Fig 8.

| **Sample** | **Sequencing protocol** | **Mutant (total cov/bp)** | **WT (total cov/bp)** |
| --- | --- | --- | --- |
| radA_mito | nanopore | 222.9 | 101.6 |
| recA3_mito | nanopore | 736.4 | 372.1 |
| why2_mito | nanopore | 170.9 | 82.5 |
| msh1_mito | nanopore | 161.9 | 36.9 |
| radA_mito | duplex | 1600.2 | 1377.4 |
| recA3_mito | duplex | 941.4 | 647.8 |
| why2_mito | duplex | 1058.7 | 1235.2 |
| msh1_mito | duplex | 1020.9 | 1209.6 |
| msh1_plastid | nanopore | 1650.1 | 762.0 |
| osb2_plastid | nanopore | 6862.2 | 1663.7 |
| radA_plastid | nanopore | 5514.3 | 1668.5 |
| recA1_plastid | nanopore | 2422.6 | 2603.1 |
| msh1_plastid | duplex | 1754.4 | 1645.3 |
| osb2_plastid | duplex | 3244.9 | 3009.4 |
| radA_plastid | duplex | 3444.6 | 2205.6 |
| recA1_plastid | duplex | 1606.6 | 1650.2 |

**APPENDIX FOR SUPPLEMENTARY FILES**

**FileS1_mutation_counts:** Coverages, mutation counts, and variant frequencies from the Duplex Sequencing analysis of data generated in this study and in Wu *et al.,* 2020.

**FileS2_repeat_recomb_freq_mito:** Counts of recombined reads and total repeat spanning reads used to calculate repeat specific recombination frequencies. We focused our mitochondrial analysis on repeats which has at least 10 recombined reads (across all replicates)**.**

**FileS3_repeat_recomb_freq_plastid:** Counts of recombined reads and total repeat spanning reads used to calculate repeat specific recombination frequencies. We focused our plastid analysis on repeats which has at least 3 recombined reads (across all replicates)**.**
